# Expression, purification, and inhibition profile of dihydrofolate reductase from the filarial nematode *Wuchereria bancrofti*

**DOI:** 10.1371/journal.pone.0197173

**Published:** 2018-05-22

**Authors:** Andrew M. Tobias, Dea Toska, Keith Lange, Tyler Eck, Rohit Bhat, Cheryl A. Janson, David P. Rotella, Ueli Gubler, Nina M. Goodey

**Affiliations:** Department of Chemistry and Biochemistry, Montclair State University, Montclair, NJ, United States of America; National Research Council, ITALY

## Abstract

Filariasis is a tropical disease caused by the parasitic nematodes *Wuchereria bancrofti* and *Brugia malayi*. Known inhibitors of dihydrofolate reductase (DHFR) have been previously shown to kill *Brugia malayi* nematodes and to inhibit *Brugia malayi* DHFR (*Bm*DHFR) at nanomolar concentrations. These data suggest that *Bm*DHFR is a potential target for the treatment of filariasis. Here, protocols for cloning, expression and purification of *Wuchereria bancrofti* DHFR (*Wb*DHFR) were developed. The Uniprot entry J9F199-1 predicts a 172 amino acid protein for *Wb*DHFR but alignment of this sequence to the previously described *Bm*DHFR shows that this *Wb*DHFR sequence lacks a crucial, conserved 13 amino acid loop. The presence of the loop in *Wb*DHFR is supported by a noncanonical splicing event and the loop sequence was therefore included in the gene design. Subsequently, the K_M_ for dihydrofolate (3.7 ± 2 μM), *k*_cat_ (7.4 ± 0.6 s^-1^), and pH dependence of activity were determined. IC_50_ values of methotrexate, trimethoprim, pyrimethamine, raltitrexed, aminopterin, (-)-epicatechin gallate, (-)-epicatechin, and vitexin were measured for *Wb*DHFR and *Bm*DHFR. Methotrexate and structurally related aminopterin were found to be effective inhibitors of *Wb*DHFR, with an K_I_ of 1.2 ± 0.2 nM and 2.1 ± 0.5 nM, respectively, suggesting that repurposing of known antifolate compound may be an effective strategy to treating filariasis. Most compounds showed similar inhibition profiles toward both enzymes, suggesting that the two enzymes have important similarities in their active site environments and can be targeted with the same compound, once a successful inhibitor is identified.

## Introduction

Lymphatic filariasis (elephantiasis) is a disfiguring and incapacitating disease caused by three species of mosquito borne parasitic worms, *Wuchereria bancrofti*, which is responsible for 90% of the cases, *Brugia malayi and Brugia timori*. This disease threatens the well-being of 947 million people in 54 countries. Clinical manifestations include lymphedema of the limbs (currently approximately 15 million cases worldwide) and hydrocele (swelling of the scrotum and penis, approximately 25 million cases) [1, 2]. Those infected with filariasis further suffer from stigma, disabilities, and the associated economic consequences.

Dihydrofolate reductase (DHFR) is an NADPH dependent enzyme that catalyzes the formation of tetrahydrofolate from dihydrofolate [3, 4]. With the exception of some prokaryotes[5], DHFR is a ubiquitous enzyme required for folate metabolism and DNA synthesis. As such, DHFR inhibition by “antifolates” has proven to be a successful strategy in the treatment of cancer, bacterial infections and malaria [6, 7]. A recent *Brugia malayi* DHFR (*Bm*DHFR) 3-D structural modeling and docking analysis predicted several antifolate compounds to be effective inhibitors of the enzyme [8]. These predictions are potentially supported by findings reported in three recent articles that show *Brugia malayi* nematode mobility decreased in the presence of antifolate agents [9–11]. Moreover, folic acid reversal studies have shown that the mobility of microfilariae decreased less when the nematodes were pre-incubated with folic acid before treatment with the antifolate compounds. Hande and coworkers also predicted vitexin, a compound found in passion flower, and the green tea compounds epicatechin and (-)-epicatechin gallate to be inhibitors of *Bm*DHFR [8].

We recently developed methods to clone, express and purify *Bm*DHFR, and have demonstrated its inhibition by well-known antifolates [12]. DHFR from *Wuchereria bancrofti* (*Wb*DHFR) is 96% identical to *Bm*DHFR in amino acid sequence. We now report the development of methods to clone, express and purify *Wb*DHFR and compare its kinetic parameters and inhibitor profile to those of *Bm*DHFR. Such a comparison allows insights into whether the amino acid differences between the two sequences have impact on kinetic parameters and inhibitor binding.

## Methods

### *Wuchereria bancrofti* (*Wb*) gene sequence development

A nucleotide sequence encoding *Wb*DHFR with an N-terminal His-6 tag was designed, synthesized, and codon optimized for expression in *E*. *coli* by Genewiz. The resulting DHFR gene sequence was subcloned into pET25b via NdeI and XhoI sites and transformed into the *E*. *coli* LOBSTR strain for protein expression.

### Expression and purification of *Wb*DHFR

*Wb*DHFR was expressed at 25°C in LB media with 100 μg/mL ampicillin and induction overnight with IPTG at 0.3 mM. The enzyme was harvested by centrifuging the *E*. *coli* mixture at 5,000 rpm for 30 min at 4°C using a JA-10 rotor in a Beckman Avanti J-26S XP centrifuge. The pellet was collected and supernatant discarded. This pellet was then resuspended using equilibration buffer (10 mM imidazole, 20 mM Na_2_HPO_4_, 300 mM NaCl, 0.1 mM DTT, at pH 7.4) and soluble protein prepared by sonication of the wet cell paste followed by centrifugation of the mixture using a Sorvall ST16R centrifuge at 5,000 rpm for 30 min at 4°C. The supernatant, rich in soluble *Wb*DHFR, was collected and pellet discarded. His-tagged *Wb*DHFR was purified at pH 7.4 using Ni-NTA resin. The column was washed with 100 mM imidazole wash buffer (100 mM imidazole, 20 mM Na_2_HPO_4_, 300 mM NaCl, 0.1 mM DTT, at pH 7.4) before being eluted with 250 mM imidazole elution buffer (250 mM imidazole, 20 mM Na_2_HPO_4_, 300 mM NaCl, 0.1 mM DTT, at pH 7.4). Protein was concentrated, and the buffer was exchanged to 20 mM Na_2_HPO_4_, 300 mM NaCl, at pH 7.4 and the concentration was determined spectroscopically at 280 nm using the extinction coefficient 25,440 M^-1^cm^-1^.

### Enzymatic activity assays

To characterize DHFR enzymatic activity, we measured absorbance at 340 nm to follow the disappearance of DHF substrate and NADPH cofactor over time [12]. The K_M_ of *Wb*DHFR for DHF was determined over a concentration range of 3.8 to 195 μM DHF, at 25°C, in MTEN buffer (50 mM 2-morpholinoethane sulphonic acid (MES), 25 mM Tris, 25 mM ethanolamine, 100 mM NaCl, and 1mM DTT) at pH 6.0. Initial velocity was plotted as a function of DHF concentration using KaleidaGraph and the Michaelis-Menten equation was fitted to the data. Catalytic activities of *Wb*DHFR and *Bm*DHFR were determined at various pH values (5.5–9.0) in MTEN buffer. The MTEN buffer used for all the reported assays has essentially a constant ionic strength at 0.15 over the pH range for which pH values were measured [13, 14]. Initial velocity was plotted as a function of pH using Excel.

### Inhibition studies

Previous computational research predicted some green tea compounds to be inhibitors of *Bm*DHFR [8]. Compounds (-)-epicatechin, (-)-epicatechin gallate, and vitexin were tested as inhibitors of *Wb*DHFR and *Bm*DHFR. The compounds (-)-epicatechin and (-)-epicatechin gallate were synthesized as described previously [15]. Additionally, methotrexate, trimethoprim, pyrimethamine, aminopterin, and raltitrexed were tested as inhibitors of *Wb*DHFR and *Bm*DHFR. Stock solutions of aminopterin and raltitrexed were prepared in water and stock solutions of the other drugs were prepared in DMSO. Control experiments were conducted to confirm that 5% DMSO (final concentration in the experimental wells) did not affect *Wb*DHFR and *Bm*DHFR activity (data not shown). The concentrations of methotrexate and aminopterin were determined spectroscopically in 0.1 M NaOH at 302 nm using an extinction coefficient of 22,700 M^-1^cm^-1^. Enzyme activity was measured in wells (200 μL) with 12.5 nM *Wb*DHFR or 40 nM *BmDHFR* and 100 μM NADPH and 50 μM DHF in MTEN buffer at pH 6.0 at 25°C. Disappearance of DHF and NADPH was observed by measuring absorbance at 340 nm to measure the DHFR activity in a SpectraMax M3 microplate reader. For active inhibitors, IC_50_ curves were generated using KaleidaGraph and the IC_50_ values were obtained by fitting the data to the Hill equation with Hill coefficient, *n*_*H*._ = 1. All experiments were completed in triplicate.

To determine the mechanism of inhibition, Dixon plots were created and analyzed for the inhibitors against *Wb*DHFR. We determined *Wb*DHFR activity as described above at DHF concentrations of 2 μM, 4 μM, and 8 μM in 200 μL reaction volumes. *Wb*DHFR (6 nM) and 100 μM NADPH cofactor were included in 1 X MTEN buffer at pH 6.0. The concentrations of inhibitors in the assay were: methotrexate (0, 3.125 nM, 6.25 nM, and 12.5 nM), trimethoprim (0, 10 μM, 20 μM, and 40 μM), pyrimethamine (0, 17.5 μM, 35 μM, and 70 μM), aminopterin (0, 3 nM, 6 nM, 12 nM), and raltitrexed (0, 5 μM, 10 μM, and 20 μM). The reciprocal initial velocities were plotted against inhibitor concentration for each substrate in Excel to create the Dixon plots. Substrate trend-lines were extended to calculate the intersection point,–K_I_. Each Dixon plot was generated in triplicate.

## Results

### Design and subcloning of a *Wb*DHFR gene into a bacterial expression vector

The Uniprot entry J9F199-1 predicts a 172 amino acid DHFR protein for *Wb*. Alignment of this sequence to the previously described *Bm*DHFR [12], however, shows that this *Wb*DHFR sequence lacks a crucial 13 amino acid loop that is conserved across a number of DHFR proteins from different species (data not shown). The presence of the loop in *Wb*DHFR can be supported by a noncanonical splicing event (data not shown) and the loop sequence was therefore included in the gene design.

### Expression and purification of *Wb*DHFR

**Fig 1:**
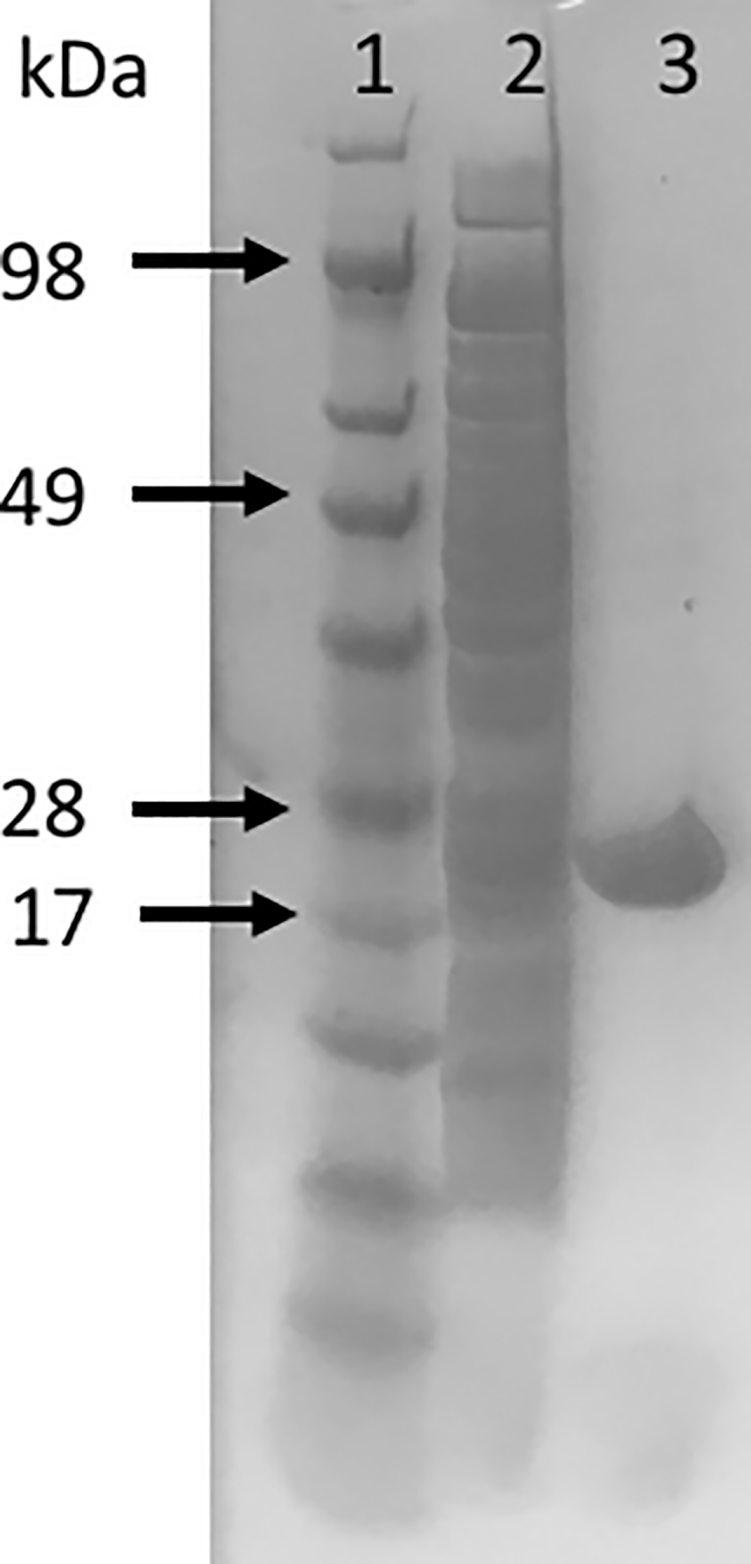
SDS-PAGE gel of *Wb*DHFR protein after purification and concentration. Lane 1: SeeBlue Plus2 Pre-stained Protein Standard (Novex); Lane 2: Column flow through; Lane 3: Purified *Wb*DHFR (8.25 μg).

To make *in vitro* studies of *Wb*DHFR possible, a protocol was developed for expression and purification of *Wb*DHFR using Ni-NTA resin; approximately 0.9 mg protein / 1 L of culture was obtained (Fig 1). Attempting to purify *Wb*DHFR using the protocol previously developed for *Bm*DHFR[12] resulted in protein with larger molecular weight impurities. To obtain *Wb*DHFR of increased purity, the imidazole concentration in the wash buffer was changed from 25 mM to 100 mM. With this modification, we were able to successfully purify *Wb*DHFR (Fig 1).

### Kinetic characterization of *Wb*DHFR

**Fig 2:**
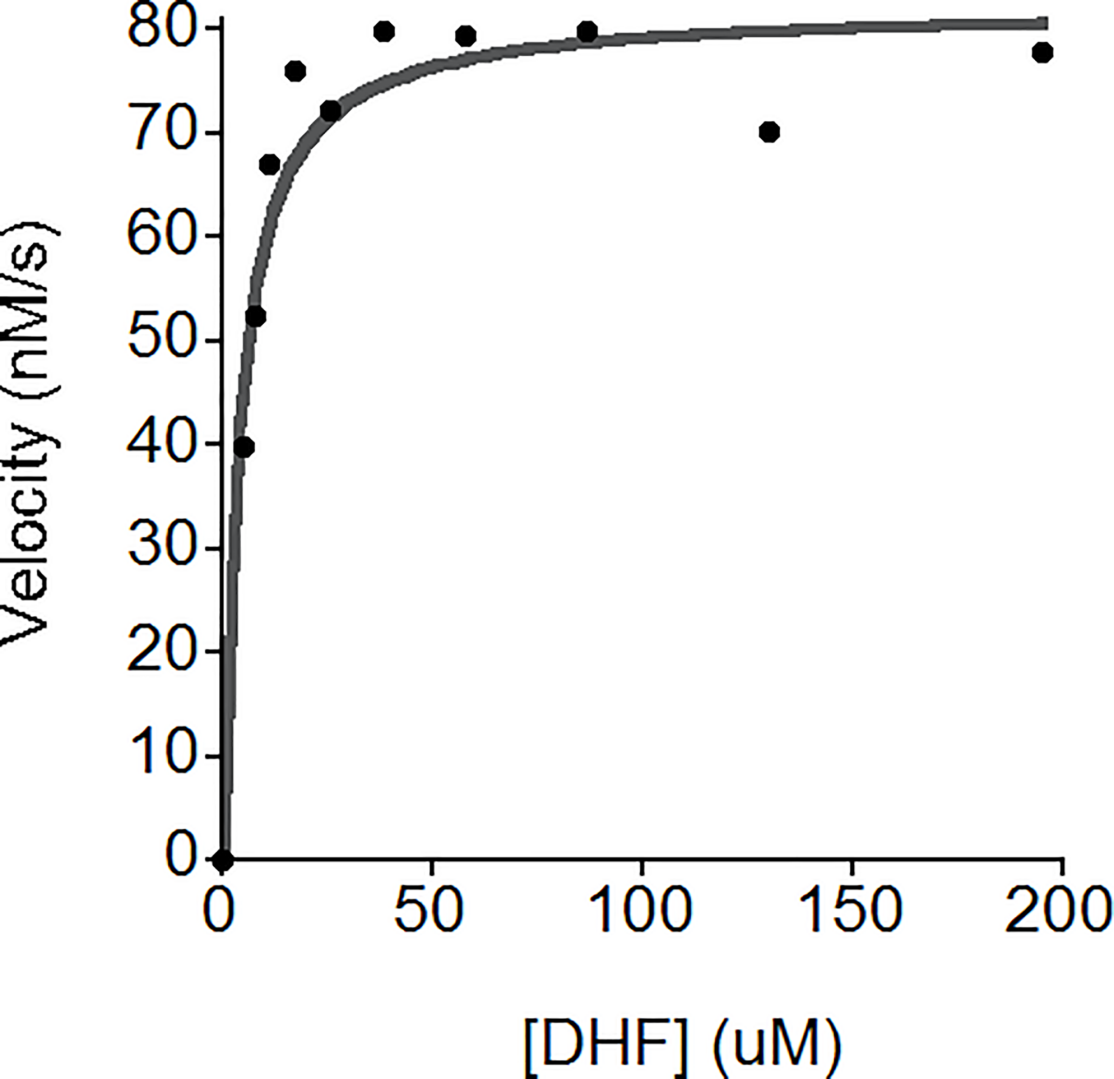
Representative Michaelis-Menten curve. The conditions in the experimental wells (200 μL) were 100 μM NADPH, 12.4 nM *Wb*DHFR in 1 X MTEN buffer at pH 6.0 with DHF concentrations ranging from 0 to 195 μM. The Michaelis-Menten equation was fitted to the data using KaleidaGraph. The Michaelis-Menten constant for DHF was determined by averaging values from fitting three separate data sets and found to be 3.7 ± 2.0 μM (S.D.).

**Fig 3:**
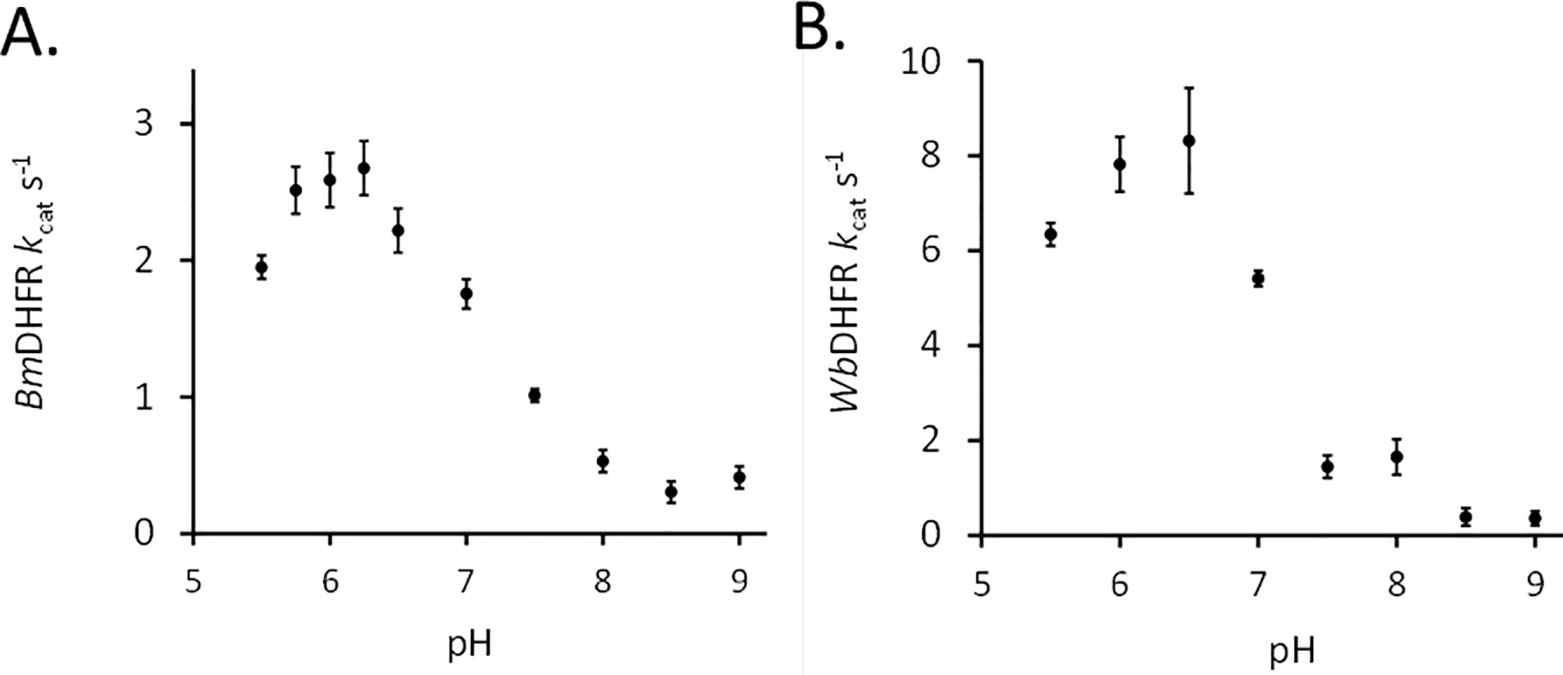
**The pH based activity curve for *Wb*DHFR (A.) and *Bm*DHFR (B.).** Enzyme concentrations in the wells were 11 nM *Wb*DHFR and 37 nM *Bm*DHFR. The wells also contained 100 μM NADPH, 100 μM DHF in 1 X MTEN buffer with pH values ranging from 5.5 to 9.0.

Kinetic characterization of *Wb*DHFR revealed a catalytic activity of 7.4 ± 0.6 s^-1^ (S.E) at pH 6. This *k*_cat_ is higher than what was found for *Bm*DHFR, 2.2 ± 0.2 s^-1^ (S.E.), at the same pH value. The K_M_ found for DHF and *Wb*DHFR, 3.7 ± 2.0 μM (S.D., Fig 2), is lower compared to the K_M_ value previously determined for *Bm*DHFR (14.7 ± 3.6 μM); data for individual trials is included in S1 Table [12]. The activity versus pH profile of *Wb*DHFR was found to be similar to that of *Bm*DHFR (Fig 3). The different y-axis values in the two profiles indicate that *Wb*DHFR catalyzes the reaction faster than *Bm*DHFR at optimal pH values.

**Fig 4:**
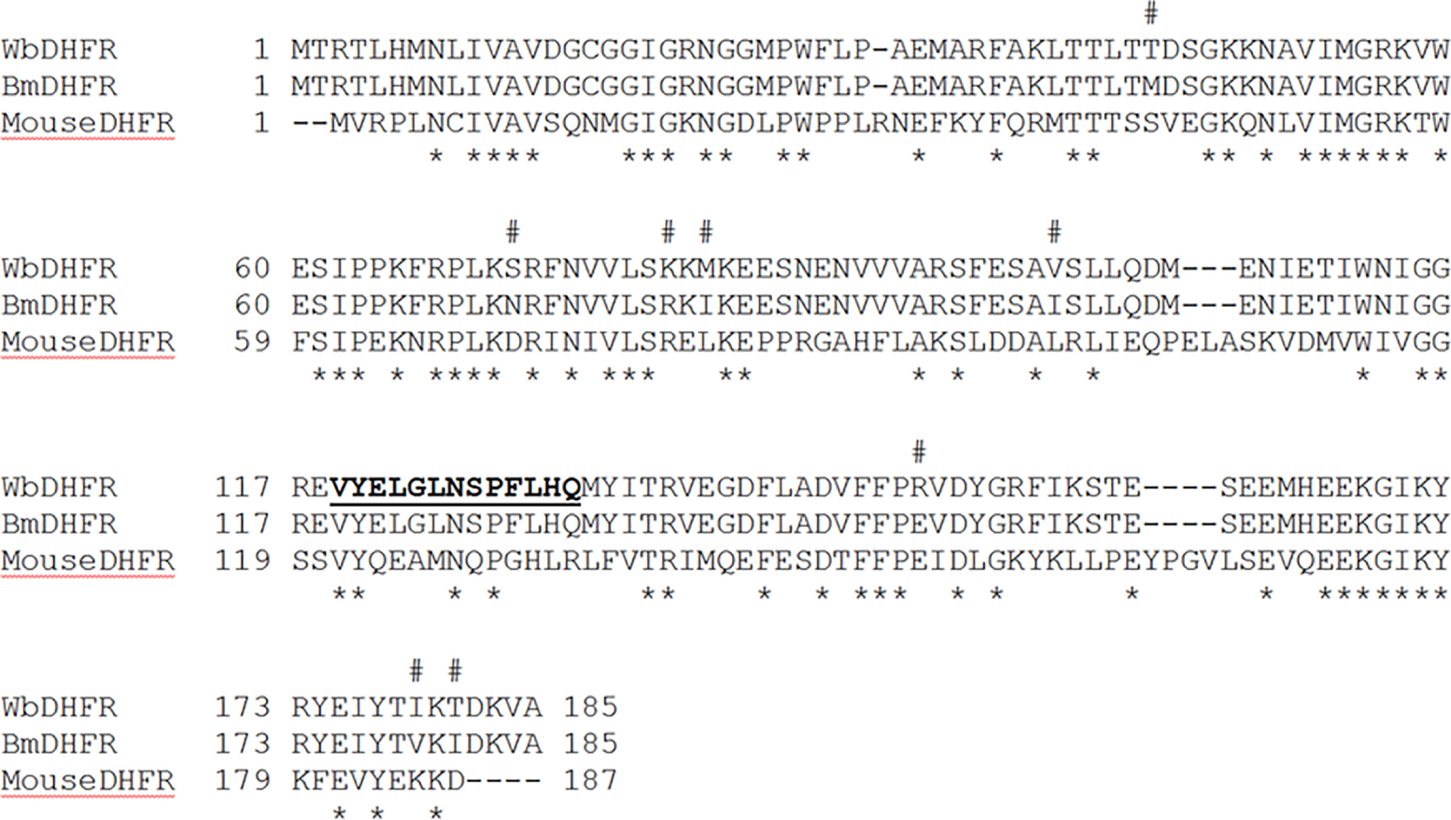
CLUSTAL alignment between *Wb*, *Bm* and mouse DHFRs. The eight amino acid differences between *Wb* and *Bm*DHFR are marked with #. The 13 amino acid region missing from the Uniprot entry and designed into the gene construct based on homology to the *Bm*DHFR is shown in bold type and underlined. The *s denote identical residues conserved among the three sequences.

*Wb*DHFR and *Bm*DHFR have similar but not identical steady-state kinetic characteristics. Comparison of the *Wb*DHFR and *Bm*DHFR amino acid sequences shows eight residues to be different (Fig 4). There are no crystal structures available for either *Wb*DHFR or *Bm*DHFR and we therefore cannot directly examine the location of the residues with different sidechains. Supporting information shows the locations of the corresponding residues superimposed on a mouse DHFR structure (PDB# 1U70) (S1 Fig), which is the DHFR with the highest level of sequence identity to *Wb*DHFR and *Bm*DHFR and an available solved structure.

### Inhibition profile of *Wb*DHFR and *Bm*DHFR

**Fig 5:**
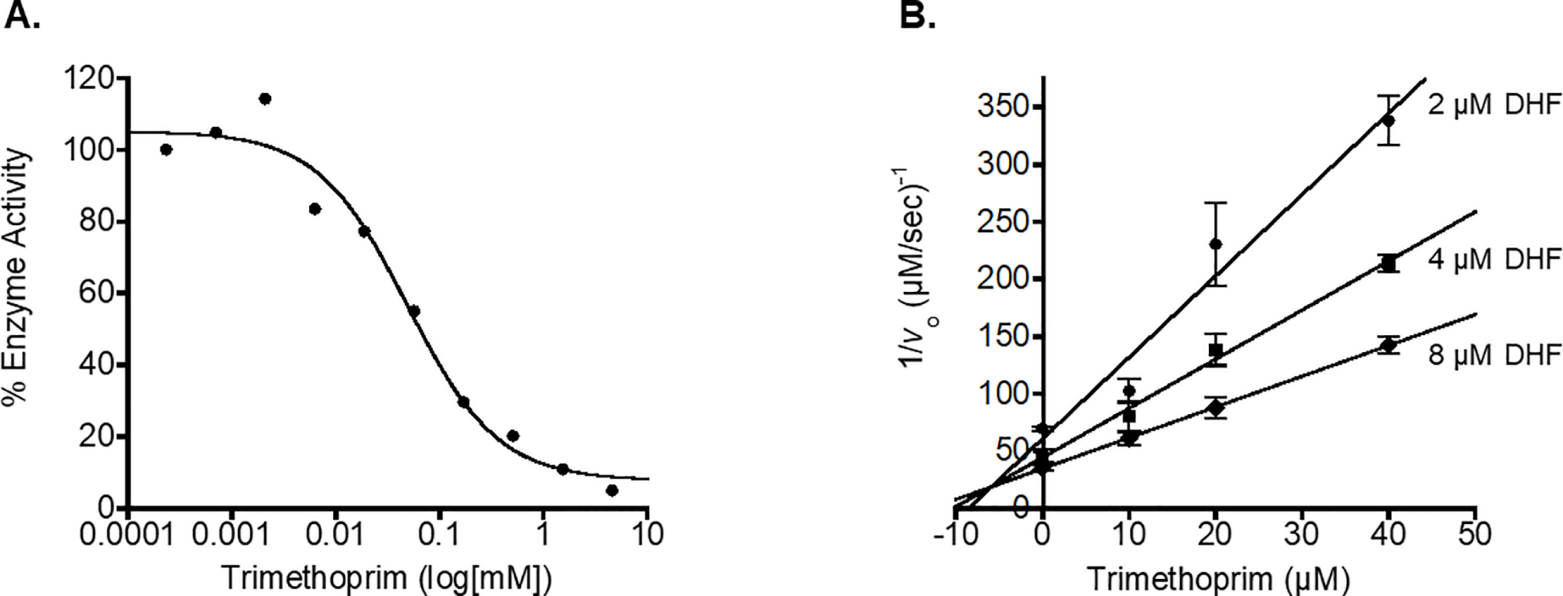
**Representative IC_50_ curve (A.) and Dixon plot (B.) for trimethoprim.** The inhibition experiments were carried out at 25°C in 1 X MTEN buffer at pH 6.0. The *Wb*DHFR activity was assessed by monitoring the disappearance of NADPH and DHF at 340 nm over time. To obtain the IC_50_ (Panel A.), 100 μM NADPH, 50 μM DHF, 12 nM *Wb*DHFR, and trimethoprim ranging from 0.2 nM to 4.7 mM were mixed in a total volume of 200 μL. The experiment was conducted in triplicate and a representative plot is shown. The Hill Equation was used to determine the IC_50_ values for trimethoprim and the average of the three values was 83 ± 25 μM. The Dixon plot (Panel B.) was generated by evaluating the initial velocity against varying concentrations of Trimethoprim and DHF. DHF concentrations of 2 μM, 4 μM, and 8μM and trimethoprim concentrations of 0 μM, 10 μM, 20 μM, and 40 μM were used in the assays. The velocities for each DHF concentration were plotted and the K_I_ was calculated to be 6 ± 0.06 μM (S.D.).

We determined IC_50_ values for several known antifolate and green tea compounds against *Bm*DHFR and *Wb*DHFR using the Hill Equation in KaleidaGraph (Table 1); data for individual values is shown in S2 Table [16]. The data show that methotrexate, trimethoprim, raltitrexed, pyrimethamine, and aminopterin inhibit *Wb*DHFR. We did not observe inhibition for (-)-epicatechin gallate, (-)-epicatechin, or vitexin against either *Wb*DHFR or *Bm*DHFR. We used Dixon plots to experimentally investigate whether the five compounds that show inhibition based on IC_50_ values (Table 1) act as competitive inhibitors for *Wb*DHFR. We plotted the reciprocals of the initial velocity at different substrate concentrations against inhibitor concentrations. A linear equation was fitted to the data at each substrate concentration. The resulting lines for all inhibitors tested crossed in the top left quadrant, indicating a competitive inhibition mechanism (Fig 5 and S3 Fig) [17]. The negative x-axis values of the point of intersection of the lines for all pairs of individual lines were determined and the average of these values was used to obtain the K_I_ values listed in Table 1; each experiment was conducted in triplicate and the standard deviations are shown and values from individual trials are shown in S3 Table. The K_I_ values for *Bm*DHFR in Table 1 were obtained using the Cheng-Prusoff Equation [18]. For the two tight-binding inhibitors aminopterin and methotrexate against *Bm*DHFR, a modification of the Cheng-Prusoff Equation for competitive inhibition for tightly bound inhibitors was needed and we report an upper limit for the K_I_ values [19, 20]. Inhibitor structures are shown in supporting information (S2 Fig). Most inhibitors that were tested have similar IC_50_ and K_I_ values towards both nematode homologs but pyrimethamine inhibits *Bm*DHFR with a K_I_ value of 3.6 ± 1.5 μM while this drug binds *Wb*DHFR four-times less tightly with a K_I_ of 15 ± 6 μM. These data suggest similarities but also subtle differences in the active sites of the two enzymes that have only eight different amino acids in their sequences.

**Table 1:** IC_50_ values for compounds tested against *Bm*DHFR and *Wb*DHFR.

	IC_50_ (μM)		K_I_ (μM)	
Compounds	*Bm*DHFR	*Wb*DHFR	*Bm*DHFR	*Wb*DHFR
Methotrexate	0.0022 ± 0.0014	0.018 ± 0.003	<0.0005 ± 0.0003	0.0007 ± 0.0001
Trimethoprim	65 ± 13	83 ± 25	15 ± 3	5.98 ± 0.06
Raltitrexed	7.3 ± 0.2	18 ± 10	1.6 ± 0.04	2 ± 1
Pyrimethamine	16 ± 7	454 ± 37	3.6 ± 1.5	15 ± 6
Aminopterin	0.0075 ± 0.0003	0.014 ± 0.005	<0.0017 ± 0.0001	0.0021 ± 0.0005
(-)-Epicatechin gallate	>1000	>1000	NA	NA
(-)-Epicatechin	>2500	>2500	NA	NA
Vitexin	>240	>240	NA	NA

The values are averages from triplicates with standard deviations shown. The experiments for each compound and enzyme were conducted at pH 6.0, at room temperature, with 100 μM NADPH, 50 μM DHF, and 40 nM *Bm*DHFR or 12.1 nM *Wb*DHFR in a side-by-side format, using the same solutions. The IC_50_ values were obtained using the Hill Equation in KaleidaGraph. The K_I_ values for *Wb*DHFR were obtained from Dixon plots (Fig 5 and S3 Fig) by evaluating the initial velocity against varying concentrations of inhibitor and DHF. The K_I_ values for *Bm*DHFR were obtained using the Cheng-Prusoff Equation.

The IC_50_ value determined here for pyrimethamine for *Bm*DHFR is different compared to the previously determined value of the same drug against the same enzyme: 15.6 ± 6.6 μM found now versus 109 ± 34 μM found previously [12]. To verify the drug stock concentration in the current study, the extinction coefficient for pyrimethamine was determined to be 6.7 ± 0.8 mM^-1^ cm^-1^ at 268 nm in 1 X MTEN at pH 6.0.

### Comparison of current data to previous computational predictions

The data agrees with some of the computational predictions by Hande and coworkers [8]; for example, they authors predicted that trimethoprim would inhibit *Bm*DHFR with a K_I_ of 11 μM and we found the K_I_ of trimethoprim to be 15 μM against *Bm*DHFR. On the other hand, vitexin was predicted to be a 465 nM inhibitor of *Bm*DHFR, but in our assays we did not observe any inhibition for vitexin against *Bm*DHFR (Table 1). Similarly, (-)-epicatechin and (-)-epicatechin gallate were predicted to have K_I_ values of 76 μM and 48 μM against *Bm*DHFR[8] but neither compound showed any inhibitory activity against *Bm*DHFR or *Wb*DHFR, even at concentrations greater than 10 mM. We also examined other compounds that are structurally related to (-)-epicatechin and (-)-epicatechin gallate and observed similar results. As the authors state themselves, the computational predictions must be interpreted with caution due to a lack of a crystal structure for any of the filarial parasite DHFRs.

## Discussion

We found that the Uniprot entry J9F199-1 for *Wb*DHFR lacks a crucial 13 amino acid loop. *Wb*DHFR, consisting of 185 amino acids (Fig 4), was successfully designed and subcloned into the pET25b expression vector and expressed in LOBSTR *E*. *coli* cells using a modified version of a protocol previously developed for *Bm*DHFR. The methods that were developed to purify active *Wb*DHFR for *in vitro* studies will facilitate the testing of additional antifolate compounds as potential inhibitors in the treatment of filariasis.

Well known antifolates, methotrexate and trimethoprim, were found to inhibit *Wb*DHFR with K_I_ values of 1.2 ± 0.2 nM and 6 ± 0.06 μM, respectively. These K_I_ values are significantly different from those of methotrexate and trimethoprim against human DHFR (40 pM and 1.38 μM, respectively) [21], indicating that there are differences in the inhibitor binding of the human DHFR compared to the parasite homologs that will likely enable discovery of selective inhibitors. These data suggest that repurposing of known antifolate compounds can be an effective approach for the treatment of filariasis. The expression, purification and basic kinetic analysis of *Wb*DHFR we publish here make it possible to test other synthetic molecules proven to act on DHFRs from other organisms as inhibitors of *Wb*DHFR. *Bm*DHFR and *Wb*DHFR have similar kinetic and inhibition parameters; 177 of the 185 amino acid residues are conserved (Fig 4, S1 Fig). We are currently working toward obtaining an x-ray crystal structure of *Wb*DHFR with an inhibitor and NADPH bound. Such a structure will further facilitate the development of antifolate compounds in the treatment of filariasis. Most of the antifolates that were tested, including those with lower IC_50_ values, inhibit the two homologs similarly, suggesting the possibility that one DHFR inhibitor could be used to treat both filarial parasites. Such an approach would be helpful in resource-poor settings where the infrastructure to determine which parasitic infection is present is not available.

## Supporting information

S1 Fig Cartoon structure of mouse DHFR (PDB # 1U70). Cofactor NADPH (left) and methotrexate (right) are shown as black lines. Methotrexate is located in the inhibitor binding site. The residue positions corresponding to those positions that have different amino acid residues present in the *Bm*DHFR and *Wb*DHFR sequences are indicated as black spheres (See Fig 4 of the research article). Numbering of these residues in the figure is based on mouse DHFR sequence. This figure was created using Chimera.(Pettersen E.F., et. al. 2004. UCSF Chimera—a visualization system for exploratory research and analysis. *J*. *Comput*. *Chem*. *13*, 1605–12.)
(TIF)

S2 Fig Structures of compounds tested as inhibitors against *Wb* and *Bm*DHFR enzymes. Structures were drawn with ChemDraw.
(TIFF)

S3 Fig **Dixon Plots for methotrexate (A.), raltitrexed (B.), pyrimethamine (C.), and aminopterin (D.) for *Wb*DHFR.** All reactions were performed at 25°C in 1 X MTEN buffer at pH 6.0. The concentration of WbDHFR and NADPH were kept constant at 6 nM and 100 μM, respectively. DHF concentrations of 2, 4, and 8 μM were used. All experiments were performed in triplicate. The plots were generated in Excel. The K_I_ values are shown in S1 Table. Data for trimethoprim is shown in Fig 5.
(TIF)

S1 Table Michaelis-Menten constant K_M_ and *k*_cat_ values for *Wb*DHFR at pH 6.0 from individual trials. (DOCX)

S2 Table IC_50_ values for compounds tested against *Wb*DHFR (top) and *Bm*DHFR (bottom) from each trial. (DOCX)

S3 Table K_I_ values for compounds tested against *Wb*DHFR from individual trials. (DOCX)
