# Supplementary figures and images for: Expression, purification, and inhibition profile of dihydrofolate reductase from the filarial nematode Wuchereria bancrofti
Source: PLoS One. 2018 May 22;13(5):e0197173. doi: 10.1371/journal.pone.0197173 (PMC5963757; doi:10.1371/journal.pone.0197173)

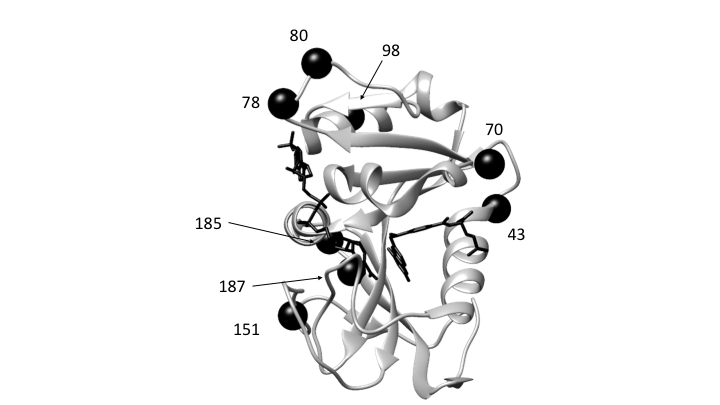

Supplement: S1 Fig — Cofactor NADPH (left) and methotrexate (right) are shown as black lines. Methotrexate is located in the inhibitor binding site. The residue positions corresponding to those positions that have different amino acid residues present in the BmDHFR and WbDHFR sequences are indicated as black spheres (See Fig 4 of the research article). Numbering of these residues in the figure is based on mouse DHFR sequence. This figure was created using Chimera.(Pettersen E.F., et. al. 2004. UCSF Chimera—a visualization system for exploratory research and analysis. J. Comput. Chem. 13, 1605–12.) (TIF) [file pone.0197173.s001.tif]

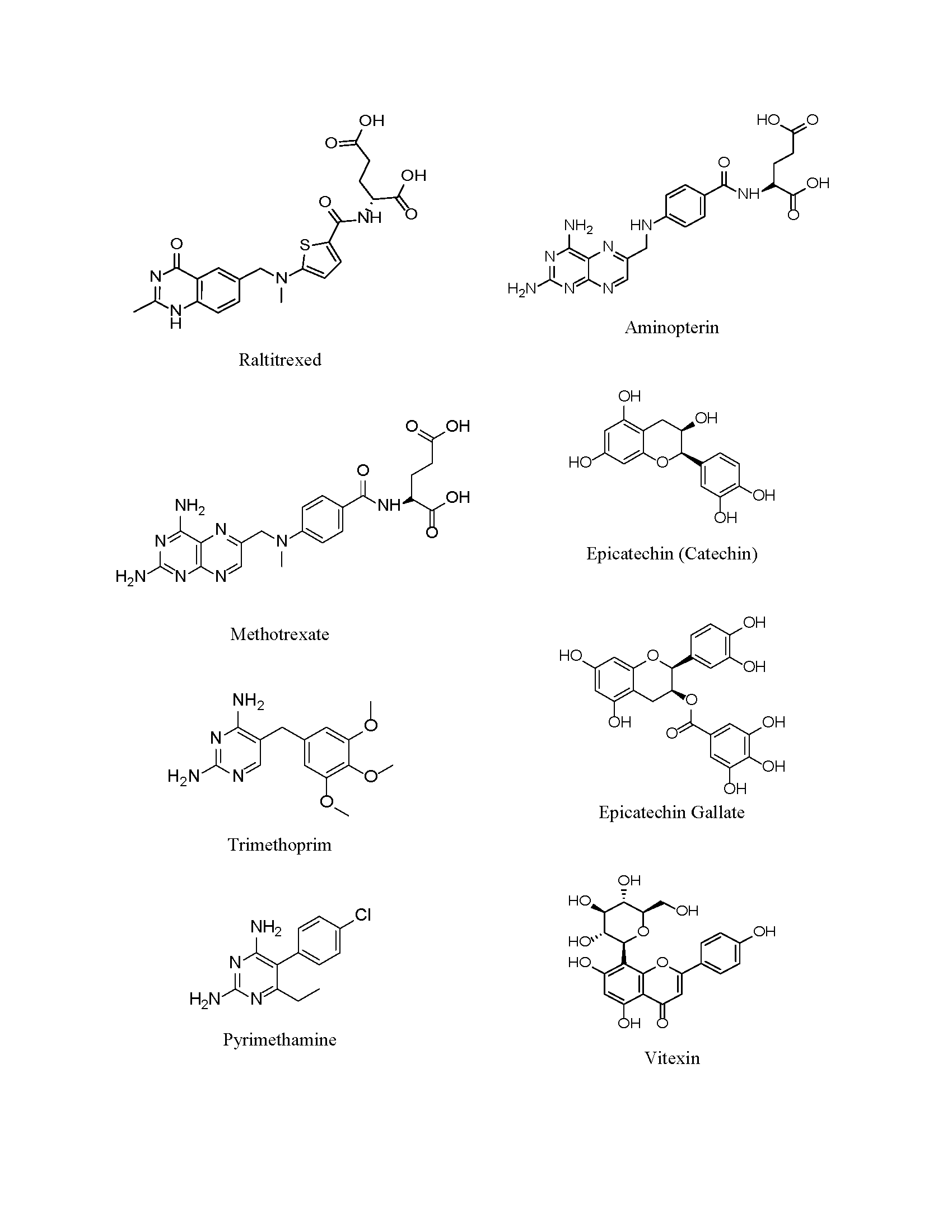

Supplement: S2 Fig — Structures were drawn with ChemDraw. (TIFF) [file pone.0197173.s002.tiff]

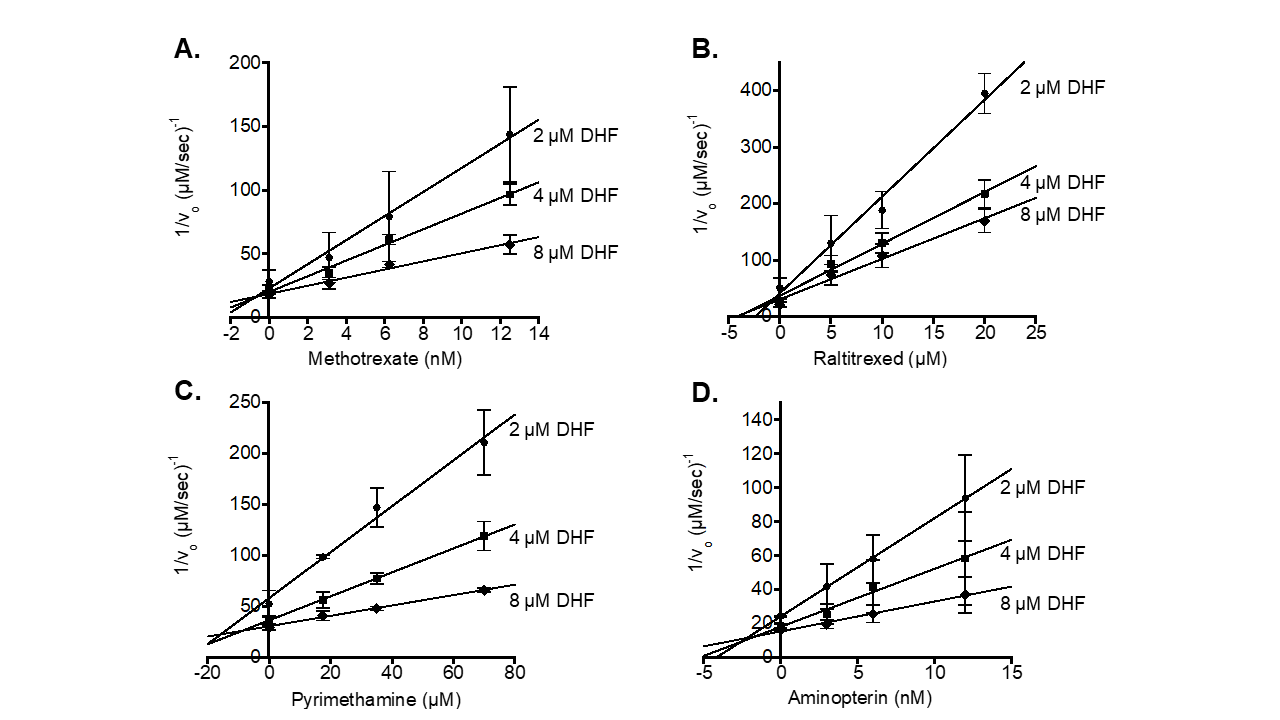

Supplement: S3 Fig — Dixon Plots for methotrexate (A.), raltitrexed (B.), pyrimethamine (C.), and aminopterin (D.) for WbDHFR. All reactions were performed at 25°C in 1 X MTEN buffer at pH 6.0. The concentration of WbDHFR and NADPH were kept constant at 6 nM and 100 μM, respectively. DHF concentrations of 2, 4, and 8 μM were used. All experiments were performed in triplicate. The plots were generated in Excel. The KI values are shown in S1 Table. Data for trimethoprim is shown in Fig 5. (TIF) [file pone.0197173.s003.TIF]
