# Supplementary material for: Expression, purification, and inhibition profile of dihydrofolate reductase from the filarial nematode Wuchereria bancrofti
Source: PLoS One. 2018 May 22;13(5):e0197173. doi: 10.1371/journal.pone.0197173 (PMC5963757; doi:10.1371/journal.pone.0197173)
Supplement: S1 Table — (DOCX) [file pone.0197173.s004.docx]

**S1 Table.** **Michaelis-Menten constant K_M_ and *k*_cat_ values for *Wb*DHFR at pH 6.0 from individual trials.**

|  | **Trial 1** | **Trial 2** | **Trial 3** | **Trial 4** | **Average** | **S.D.** |
| --- | --- | --- | --- | --- | --- | --- |
| K_M_ (μM) | 3.72 | 5.66 | 1.68 | N/A | 3.7 | 2.0 |
| *k*_cat_ (s^-1^) | 7.32 | 8.37 | 8.16 | 5.82 | 7.4 | 0.6 |
