# Supplementary material for: Expression, purification, and inhibition profile of dihydrofolate reductase from the filarial nematode Wuchereria bancrofti
Source: PLoS One. 2018 May 22;13(5):e0197173. doi: 10.1371/journal.pone.0197173 (PMC5963757; doi:10.1371/journal.pone.0197173)
Supplement: S2 Table — (DOCX) [file pone.0197173.s005.docx]

**S2 Table.** **IC50 values for compounds tested against *Wb*DHFR (top) and *Bm*DHFR (bottom) from each trial.**

| **IC50 against *Wb*DHFR** | | | | | |
| --- | --- | --- | --- | --- | --- |
|  | **Trial 1** | **Trial 2** | **Trial 3** | **Average** | **S.D** |
| Methotrexate | 15.1 nM | 20.1 nM | 17.5 nM | 17.6 nM | 3 |
| Trimethoprim | 109 µM | 90.2 µM | 49.1 µM | 82.7 µM | 25 |
| Raltitrexed | 28.6 µM | 11.7 µM | 13.0 µM | 17.8 µM | 10 |
| Pyrimethamine | 449 µM | 494 µM | 420 µM | 454 µM | 37 |
| Aminopterin | 20.3 nM | 9.96 nM | 13.0 nM | 14.4 nM | 5 |
|  | | | | | |
| **IC50 against *Bm*DHFR** | | | | | |
| Methotrexate | 1.02 nM | 1.93 nM | 3.68 nM | 2.21 nM | 1 |
| Trimethoprim | 80.4 µM | 61.1 µM | 48.9 µM | 64.7 µM | 13 |
| Raltitrexed | 7.1 µM | 6.83 µM | 13.0 µM | 7.30 µM | 0.2 |
| Pyrimethamine | 16.4 µM | 21.8 µM | 8.7 µM | 16 µM | 7 |
| Aminopterin | 7.20 nM | 7.60 nM | 7.84 nM | 7.55 nM | 0.3 |
