# Supplementary material for: Expression, purification, and inhibition profile of dihydrofolate reductase from the filarial nematode Wuchereria bancrofti
Source: PLoS One. 2018 May 22;13(5):e0197173. doi: 10.1371/journal.pone.0197173 (PMC5963757; doi:10.1371/journal.pone.0197173)
Supplement: S3 Table — (DOCX) [file pone.0197173.s006.docx]

**S3 Table.** **KI values for compounds tested against *Wb*DHFR from individual trials.**

|  | **K_I_ Trial 1** | **K_I_ Trial 2** | **K_I_ Trial 3** | **Average K_I_** | **S.D** |
| --- | --- | --- | --- | --- | --- |
| Methotrexate | 0.809 nM | 0.690 nM | 0.555 nM | 0.68 nM | 0.13 |
| Trimethoprim | 5.92 µM | 5.96 µM | 6.04 µM | 5.98 µM | 0.06 |
| Raltitrexed | 0.536 µM | 1.09 µM | 3.32 µM | 1.6 µM | 1 |
| Pyrimethamine | 20.3 µM | 15.9 µM | 8.89 µM | 15.0 µM | 6 |
| Aminopterin | 2.60 nM | 2.19 nM | 1.60 nM | 2.1 nM | 0.5 |
